# Medication knowledge, certainty, and risk of errors in health care: a cross-sectional study

**DOI:** 10.1186/1472-6963-11-175

**Published:** 2011-07-26

**Authors:** Bjoerg O Simonsen, Inger Johansson, Gro K Daehlin, Lene Merete Osvik, Per G Farup

**Affiliations:** 1 Dept. of Research, Innlandet Hospital Trust, Brumunddal, Norway; 2 Unit for Applied Clinical Research, Norwegian University of Science and Technology, Trondheim, Norway; 3 Faculty of Health, Care and Nursing, Gjoevik University College, Gjoevik, Norway; 4 Dept. of Nursing, Karlstad University, Karlstad, Sweden; 5 Dept. of Surgery, Oestfold Hospital Trust, Fredrikstad, Norway

## Abstract

**Background:**

Medication errors are often involved in reported adverse events. Drug therapy, prescribed by physicians, is mostly carried out by nurses, who are expected to master all aspects of medication. Research has revealed the need for improved knowledge in drug dose calculation, and medication knowledge as a whole is poorly investigated. The purpose of this survey was to study registered nurses' medication knowledge, certainty and estimated risk of errors, and to explore factors associated with good results.

**Methods:**

Nurses from hospitals and primary health care establishments were invited to carry out a multiple-choice test in pharmacology, drug management and drug dose calculations (score range 0-14). Self-estimated certainty in each answer was recorded, graded from 0 = very uncertain to 3 = very certain. Background characteristics and sense of coping were recorded. Risk of error was estimated by combining knowledge and certainty scores. The results are presented as mean (±SD).

**Results:**

Two-hundred and three registered nurses participated (including 16 males), aged 42.0 (9.3) years with a working experience of 12.4 (9.2) years. Knowledge scores in pharmacology, drug management and drug dose calculations were 10.3 (1.6), 7.5 (1.6), and 11.2 (2.0), respectively, and certainty scores were 1.8 (0.4), 1.9 (0.5), and 2.0 (0.6), respectively. Fifteen percent of the total answers showed a high risk of error, with 25% in drug management. Independent factors associated with high medication knowledge were working in hospitals (p < 0.001), postgraduate specialization (p = 0.01) and completion of courses in drug management (p < 0.01).

**Conclusions:**

Medication knowledge was found to be unsatisfactory among practicing nurses, with a significant risk for medication errors. The study revealed a need to improve the nurses' basic knowledge, especially when referring to drug management.

## Background

Since the report "To Err Is Human: Building a Safer Health System" was published in 2000, there has been a worldwide focus upon the risks in Health services, and how to improve patient safety [1]. Errors in medication may occur in all parts of the process from diagnosis and prescription to administration and usage. Failure to administer or incorrect dosage were the most common events reported [2]. In Norway, medication errors accounted for 27% of the adverse events reported to the Norwegian Board of Health in 2007, and for 13% of the fatal adverse events reported in the period 2001-2007 [3].

Physicians are responsible for the drug treatment, but registered nurses play an important role in carrying out the practical procedures in hospitals and community health care establishments, and have the responsibility for recognizing errors and reporting them. Therefore, adequate knowledge and ability during dispensation and administration of drugs are vital for safe drug treatment. Nurses receive their basic training in pharmacology, drug management (regulations, storage, preparation of drugs and administration to patients) and drug dose calculation from university colleges and from on-the-job training under senior nurses acting as tutors [4]. Practicing registered nurses' knowledge in medication is primarily unknown, but there have been reports of inadequate knowledge in pharmacology and drug management in some studies [5-7]. However, there is more information available about numerical skills and drug dose calculation. Nursing students and trained nurses, as well as medical students and physicians confirm that this is a complex issue [8-12]. At Norwegian university colleges, the majority of students struggle to pass a faultless drug dose calculation test which is mandatory according to the National framework for bachelor education in nursing [4]. There is, according to a recent review, insufficient evidence to suggest that deficient drug dose calculation skills are the cause of medication errors [13]. The causal relationships between knowledge, skills and risk of errors are complex and involve factors such as perceived certainty, sense of coping and self-esteem, areas that are poorly investigated [14]. All health institutions are responsible for providing personnel with a sufficient expertise in drug management, and for making sure that national legislations are followed [15].

The purpose of this study was to evaluate medication knowledge and self-reported certainty among nurses; to estimate the risk of medication errors, and explore factors associated with medication knowledge, certainty and risk of errors.

## Methods

### Participants

Registered nurses from two Norwegian hospitals with 2300 nurses, and three municipalities with 500 nurses were invited to participate in the survey. The invitation was announced in the institutions through the management line, and participants enrolled with their manager or directly with the researcher. Recruitment would be closed on reaching 200 participants.

Inclusion criteria were registered nurses with at least 1 year of work experience in a 50% part-time job or more. Nurses that were excluded were those working in outpatient clinics, those who did not administer drugs, and any who were not sufficiently fluent in Norwegian. The study was performed from September 2007 to April 2008.

### Study design

The design was a cross-sectional study performed in classrooms under controlled conditions. The participants completed a form with relevant background characteristics and performed a multiple-choice (MCQ) test in pharmacology, drug management, and drug dose calculation. The maximum time for the test was 2.5 h.

### Variables

#### Participant characteristics

The following background characteristics were recorded: age, gender, place where they grew up, place of education while studying nursing, length of work experience as a nurse in at least a 50% part-time job, employment fraction for the past 12 months, present place of work in a specific hospital department (surgery, internal medicine or psychiatry), or primary health care (nursing home or ambulatory care). In addition, further educational background was recorded: number of years of studying mathematics beyond the first mandatory year at upper secondary school; other education prior to nursing; postgraduate specialization; and further education or refresher courses in pharmacology, drug management, or drug dose calculation during the past 3 years. In addition, statements regarding sense of coping and self-esteem/wellbeing were recorded.

#### Medication knowledge and certainty

Medication knowledge was in this context used as a common term, including the disciplines of pharmacology (pharmacokinetics and dynamics), drug management (regulations, storage, preparation of drugs and administration to patients) and drug dose calculation, and each discipline consisted of different topics. The medication knowledge test was composed as a multiple-choice test, with 14 questions with 3-4 alternative answers within each discipline. The disciplines and topics were as follows (number of questions for each topic shown in brackets):

*Pharmacology*: general pharmacology (3), effects (3), side effects (3), administration formulas (2), interactions (1), and generic drugs (2).

*Drug management: *regulations (2), storage (4), dispensation (4), and administration (4).

*Drug dose calculation*: conversion of units (7), formulas for calculation of dose, quantity or strength (4), infusions (2), and dilutions (1).

The translated questions are given in Additional file 1.

To cover all the topics within each discipline, questions were put together from actual tests for bachelor nursing students at university colleges (drug dose calculation), from tests of continuing educational programs used in Norwegian hospitals, and a few questions were added based on experience from problems arising among nurses.

To assess the knowledge, the requirements to pass exams at the university colleges in Norway were used as a guideline. The limit to pass MCQ-tests is normally 60% correct answers, but for drug dose calculations, the requirement is a faultless test. For this study, nine out of 14 correct answers (64%) was chosen as the lowest acceptable score in pharmacology and drug management, and for drug dose calculations only a faultless test was accepted.

For each question, the participants indicated self-estimated certainty, graded from 0-3: 0 = very uncertain (would search for help, consult colleagues/reference books), 1 = relatively uncertain (would probably search for help), 2 = relatively certain (would probably not search for help), and 3 = very certain (would not search for help).

#### Risk of error

Risk of error was estimated by combining knowledge and certainty for each question, rated on a scale from 1 to 3 devised for this study. Correct answers combined with high certainty (relatively/very certain) was regarded as a low risk of error (score = 1), low certainty (relatively/very uncertain) independent of correct answer was regarded as a moderate risk of error (score = 2), and incorrect answer combined with high certainty (relatively/very certain) was regarded as a high risk of error (score = 3).

#### Sense of coping and self-esteem/wellbeing

Nine statements from General Health Questionnaire (GHQ 30), a Quality of life tool focusing on psychological and psychosocial symptoms, were used for the purpose of this study [16]. Five of the statements were related to coping (finding life a struggle; being able to enjoy normal activities; feeling reasonably happy; getting scared or panicky for no good reason; and being capable of making decisions), and four statements were related to self-esteem/wellbeing (overall doing things well; satisfied with the way they have carried out their task; managing to keep busy and occupied; and managing as well as most people in the same situation). The ratings of these statements were 0-3: 0 = more/better than usual, 1 = as usual, 2 = less/worse than usual, and 3 = much less/worse than usual; "as usual" was defined as the normal state.

### Ethics

The Norwegian Data Inspectorate, represented by Privacy Ombudsman for Research at Oslo University Hospital, Ullevål, approved the collection of data for the study. All participants gave written informed consent. To protect the participants from any consequences as a result of the test, data were made anonymous before analysis.

### Analyses

Comparisons between groups were analyzed with Chi-square/Fishers exact test, t-test/Mann-Witney U-test, ANOVA/Kruskal-Wallis and Pearson/Spearman tests for correlations depending on data distribution. The same tests were used for the study of associations between variables and medication knowledge, certainty and risk of error. Variables associated with medication knowledge, certainty and risk of error with p < 0.20 were included in stepwise forward linear regression analyses, corrected for age and gender, to find independent predictors. Two-tailed significance tests were used, and a p-value < 0.05 was considered statistically significant. The analyses were performed with SPSS version 13.0 (SPSS Inc., Chicago, IL, USA).

Studies testing drug dose calculation skills among nurses have shown a mean score of 75% (SD 15%), i.e. 10.5 (SD 2.1) in this study with a max score of 14 in each discipline [17-19]. This study, of 200 participants, has a power of 0.9 to detect a difference of one correct answer in two groups of the same size (p < 0.05), and was determined by the number needed for a subsequent randomized controlled study to compare two didactic methods in drug dose calculation.

Missing data were handled as described in the protocol: Unanswered questions in the medication knowledge test were scored as "incorrect answer," and unanswered certainty score as "very uncertain".

## Results

### Participant characteristics

**Figure 1:**
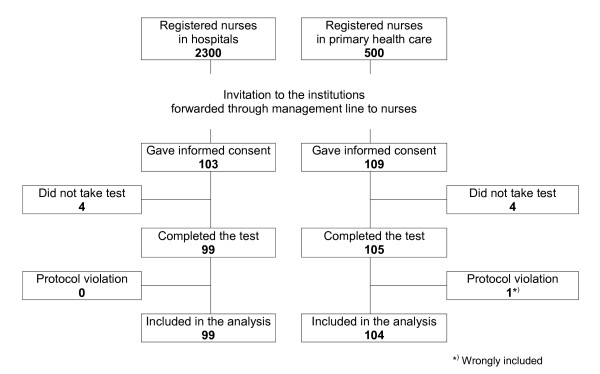
**Flow diagram of participants**.

In total, 212 registered nurses were included in the study, and 203 included in the analysis. Figure 1 shows the flow of participants from hospitals and primary health care establishments throughout the study, and Table 1 summarizes background characteristics. Demography and other characteristics were well balanced between the hospital and primary health care group, with only one exception; there were significantly more postgraduate specialists working in hospitals. Of the 99 nurses working in hospitals, 66 (67%) worked in surgery departments, including intensive care units; 25 (25%) in internal medicine wards, and 8 (8%) in psychiatry wards. In primary health care establishments, there were 52 participants from nursing homes and 52 from ambulatory health care.

**Table 1:** Characteristics of participants

	Hospitals	Primary health care	P-value
	(N = 99)	(N = 104)	
**Characteristics**			
Age in years	41.1 (8.5)	42.8 (10.1)	0.20, ns
Male gender	9 (9.1%)	7 (6.7%)	0.54, ns
Childhood outside Norway	10 (10.1%)	6 (5.8%)	0.26, ns
Education outside Norway	8 (8.1%)	3 (2.9%)	0.10, ns
Work experience as nurse in years	12.6 (8.8)	12.2 (9.7)	0.77, ns
Job size latest 12 months	0.86 (0.16)	0.86 (0.16)	0.82, ns
Mathematics beyond 1^st ^year USS^1)^	36 (36.4%)	44 (42.3%)	0.39, ns
Other education before becoming nurse	38 (38.4%)	43 (41.3%)	0.67, ns
Postgraduate specialization	47 (47.5%)	21 (20.2%)	<0.001
Relevant courses past 3 years	24 (24.2%)	22 (21.2%)	0.60, ns
GHQ - score coping^2)^	0.76 (0.28)	0.81 (0.28)	0.07, ns
GHQ - score self-esteem^2)^	1,01 (0.24)	1.02 (0.22)	0.67, ns

^1) ^Upper secondary school

^2) ^General Health Questionnaire (GHQ) score 0-3, 0 = better than usual, 1 = as usual, 2 = worse than usual, 3 = much worse than usual.

The results are given as mean (standard deviation in brackets), or number of participants (proportion in brackets).

The 80 participants (39%) with mathematics as a subject beyond the mandatory first year at upper secondary school had a median 2 years extra teaching, ranging from 1 to 4 years. Eighty-one participants (40%) had finished other education before bachelor studies in nursing; ten were auxiliary nurses and 16 had other health personnel educations (occupational therapist, pedicurist, dental assistant, health secretary, pharmacy technician, or acupuncturist). Thirty-nine participants (19%) had taken relevant courses in pharmacology, 35 (17%) in drug management, and 28 (14%) in drug dose calculations. Twenty-two participants (11%) had taken courses in all three disciplines during the past 3 years.

### Medication knowledge and certainty

Table 2 summarizes the primary outcomes for knowledge, certainty and risk of error both globally and divided by discipline. The scores for knowledge, certainty and risk of error showed statistically significant differences (p < 0.001) between the three disciplines.

**Table 2:** Primary outcomes for the MCQ test in medication knowledge, certainty evaluation and risk of error, totally and for each discipline

	Knowledge	Certainty	Risk of error
	(score 0-14)	(score 0-3)	(score 1-3)
**Total test**	29.0* (3.4)	1.9 (0.4)	1.6 (0.1)
**Pharmacology**	10.3 (1.6)	1.8 (0.5)	1.6 (0.3)
**Drug management**	7.5 (1.6)	1.9 (0.5)	1.9 (0.2)
**Drug dose calculation**	11.2 (2.0)	2.0 (0.6)	1.5 (0.3)
Statistics **	P < 0.001 ^1)^	P < 0.001 ^2)^	P < 0.001 ^2)^

The results are given as mean score (±SD)

*) score 0-42

**) Statistics refers to comparisons between the disciplines pharmacology, drug management and drug dose calculation

^1) ^ANOVA ^2) ^Kruskal-Wallis

**Figure 2:**
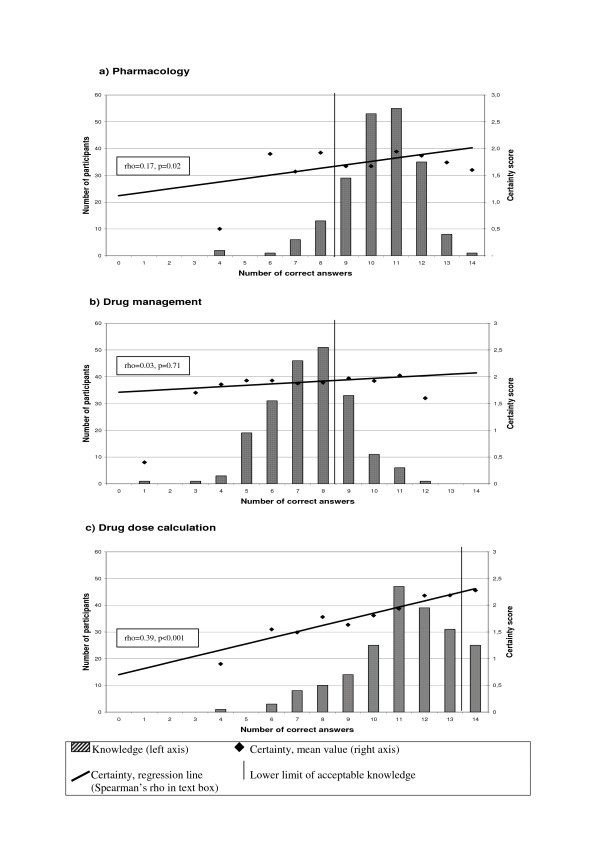
**Results of the knowledge test and certainty evaluation in the three disciplines**.

Figure 2 shows the results of the medication knowledge test and certainty evaluation in each discipline. One hundred and eighty-one (89%) scored 64% or more in pharmacology (Figure 2A) and 51 (25%) in drug management (Figure 2B). Twenty-five (12%) had a faultless test in drug dose calculations (Figure 2C).

There was a positive correlation between knowledge and certainty for pharmacology and drug dose calculations, but not for drug management. Those who had a faultless drug dose calculation test had significantly higher certainty scores (2.3) than those who failed (1.9), (p = 0.004). This was not the case for the other two disciplines (p = 0.53 and 0.42).

### Risk of error

The test results and risk of error for each discipline and topic are given in Table 3. The median number of answers with high risk of error out of the 14 questions (range in brackets) in each of the three disciplines were: pharmacology 1(0-5), drug management 4(0-10), and drug dose calculation 1(0-6), and of the total 42 questions 6(0-15).

**Table 3:** Test results and distribution of risk of error for each discipline and topic

Content (no of questions)	Correct answers	**Low risk**^**1)**^	**Moderate risk**^**2)**^	**High risk**^**3)**^
Gen. Pharmacology (3)	82%	56%	39%	5%
Effect (3)	80%	56%	41%	3%
Side effects (3)	81%	59%	34%	7%
Formulations (2)	41%	14%	45%	42%
Interactions (1)	60%	22%	68%	10%
Generics (2)	81%	68%	25%	7%

**Pharmacology (14)**	**74%**	**50%**	**39%**	**11%**

Regulations (2)	59%	46%	29%	25%
Storage (4)	33%	21%	51%	27%
Dispensation (4)	60%	48%	28%	24%
Administration (4)	63%	49%	23%	28%

**Drug management (14)**	**53%**	**40%**	**33%**	**26%**

Conversion of units (7)	76%	61%	29%	10%
Dose-quantity-strength (4)	92%	73%	24%	3%
Infusions (2)	84%	57%	40%	4%
Dilutions (1)	55%	25%	68%	8%

**Drug dose calculations (14)**	**80%**	**61%**	**32%**	**7%**

**Totalt (42)**	**69%**	**50%**	**35%**	**15%**

All percentages were calculated from the total number of answers from all 203 participants.

^1) ^Low risk: When the participant expressed relatively or very high certainty in correct answer

^2) ^Moderate risk: When the participant expressed relatively or very high uncertainty, independent of correct/incorrect answer.

^3) ^High risk: When the participant expressed relatively or very high certainty in incorrect answer.

### Factors associated with high medication knowledge, certainty and risk of error

Factors associated with high medication knowledge, certainty and risk of error are given in Table 4. Working in hospitals resulted in being the most important factor for both high medication knowledge and certainty. Medication knowledge was not associated with certainty (p = 0.4), and mathematical background was not associated with high drug dose calculation scores (p = 0.8), data not shown. No association was found between knowledge or certainty and wellbeing/self-esteem in the bivariable analysis.

**Table 4:** Association between medication knowledge, certainty evaluation and risk of error and participants' background characteristics

Associating factors	Medication knowledge (score 0-42)			Certainty (score 0-3)			Risk of error (score 1-3)		
	
	*Beta*	*P-value*	***R***^***2***^***-change***	*Beta*	*P-value*	***R***^***2***^***-Change***	*Beta*	*P-value*	***R***^***2***^***-Change***
Working in hospitals	1.80	<0.001	0.10	0.22	<0.001	0.08	-0.20	0.001	0.10
Postgraduate specialist	1.23	0.01	0.03		ns		-0.22	0.001	0.05
Course in drug management	1.60	0.006	0.03		ns		-0.21	0.004	0.03
GHQ score coping		Ns		0.06	0.005	0.04	0.05	0.005	0.03
Total adjusted R^2^			0,16			0.11			0.20

Multivariable linear regression with forward stepwise selection of variables.

## Discussion

The current study among nurses revealed that medication knowledge was insufficient, and it suggests risk for medication errors.

### Medication knowledge and certainty

The overall medication knowledge among registered nurses was lower than expected. The pharmacology test resulted as the discipline with the highest result. Although pharmacological considerations are made by physicians, some studies indicate that the importance of even this discipline may be underestimated in the education of nurses [7,20,21].

The knowledge in drug management was considered low and therefore of concern, as only one out of four participants achieved the lowest acceptable score or more. The lack of basic knowledge may be explained by little emphasis on introduction to drug management in the theoretical curriculum. This is left to the practice field, where much of the tutoring is based on master and journeyman teaching: on-the job training, without sufficient awareness of the need to connect practical techniques to theory and critical thinking [22]. The need to improve the competence in drug management was further underlined by the lack of correlation between knowledge and certainty in this discipline.

The results in drug dose calculation were also concerning, despite the fact that it was the best of the three disciplines. The positive correlation found between knowledge and certainty in this discipline indicated that the nurses were aware of their insufficient skills. The demand for faultless calculations reflects the importance of patients getting the right dose, and several studies of adverse drug events have shown that incorrect drug and doses are the most common errors [23]. The insufficient knowledge in drug dose calculations is consistent with other findings [17,18]. Nursing students face the challenges of having to complete a faultless test early on in the program, and many find mathematical problems complicated. It is likely that the difficulties of the calculations are exaggerated, and this attitude accompanies the nurses throughout their career. How to change this attitude is another discussion, not covered here.

The relatively high certainty scores supports an assumption that the most secure and self-conscious persons volunteer for such a test. The small, but statistically significant association found between certainty and sense of coping is in agreement with other studies that point out that sense of coping might be regarded as a general property, unrelated to knowledge [24].

### Risk of error

The study's focus on certainty intended to elucidate the connection between knowledge and possible risk of errors in real life. This risk was regarded as too high, since the respondents were certain that an incorrect answer was correct in 15% of the questions. In a MCQ-test, a correct answer may be a result of guessing, but the participants could highlight any uncertainty in the answer using the certainty scale. The risk of error due to pharmacological issues was small, but drug formulations were the one topic with the highest risk of error. This is important to be aware of, since nurses are responsible for switching between different generic drugs, often with different brand names and formulations. The most critical discipline for risk of error was drug management, with insufficient knowledge and no significant correlation between knowledge and certainty. Drug management is a major task for nurses, and their low basic knowledge and high estimated risk of errors give cause for concern, even if the test situation was artificial compared to real working situations. Drug dose calculation was the weakest discipline compared to requirements, but the risk of error was low. This reflects the fact that nurses often are aware of their deficient numerical skills, and do consult others.

### Factors associated with knowledge, certainty, and risk of errors

Although the factors associated with high medication knowledge, certainty and risk of error were all highly statistically significant, each of them explains just a small part of the variability of the results, and should not be over-emphasized.

The participants' working place was the most important factor associated with both high medication knowledge and certainty, and hence low risk of error. It was reassuring that working in hospitals and postgraduate specialization was associated with high knowledge, since the most advanced medical treatments take place in hospitals, and potentially harmful medication procedures are handled by specially trained nurses, both in hospitals and in primary health care establishments. Working place has also been indicated as a factor influencing doctors' ability in drug dose calculations [25].

Previous courses in general showed low association with high knowledge. Less than one in four participants had taken courses in any of the disciplines in the past 3 years, and only courses in drug management were associated with high knowledge. The information about the duration and content of the earlier courses was too limited to be able to reach a conclusion about the effect of such courses.

In contrast to the above-mentioned variables, which all were associated with a small, but statistically significant reduction in risk of error, a high sense of coping was associated with an increased risk of error. Further studies should also explore other qualities in nurses to explain a greater proportion of the variation in the test results, such as marks in maths and overall marks from upper secondary school, and educational institutions, along with other contributing factors to medication errors.

### Method strengths and limitations

MCQ-tests are increasingly used to examine knowledge and understanding in university exams. Others who have evaluated the validity of choosing three or four alternative answers have concluded that there is no difference between the two [26].

We have not found university college tests that include all disciplines. However, the questions in the present MCQ-test were adapted to the knowledge expected from the nurses, as they were composed from university colleges exams; running tests used in hospitals; and questions raised from audits. Some of the questions may seem to be of limited relevance for health personnel in primary health care, but there is an increasing demand to master administration of advanced medications outside of hospitals as well. A possible influence of different cultural backgrounds or understanding of the Norwegian language was not evaluable due to few participants with backgrounds from outside Norway. Extra years of maths was probably not a suitable indicator for high drug dose calculation knowledge; exam grades in maths would probably have been a better variable.

Self-estimated certainty compared to sense of coping and self-esteem when carrying out medication tasks was evaluated with parts of the General Health Questionnaire (GHQ 30), using 9 of the 30 statements. GHQ30 is a widely used instrument for screening mental health, developed for use in primary health care. It is a methodological weakness to use only a part of the standardized tool.

The risk estimations in the study could have benefited from a better definition of the impact of each incorrect answer. A recent review pointed out that there is no established link from miscalculations by nurses with medication errors [12]. Although the estimated risk of error in the study is not automatically transferable to medication errors in real life, it is regarded as suitable to point out risk areas that reflect reality. Risk of error was interpreted conservatively, e.g. when respondents were confident that a wrong answer was correct.

Participant selection could be a limitation of the study, and affect the external validity of the findings. Information is not available for how many of the 2800 registered nurses actually received the invitation to participate, and thus had the opportunity to volunteer for the study. But if we assume that only the most confident persons volunteered for such an examination, it is rather discouraging that, in reality, the level of knowledge is probably even lower than this study shows. However, the study population matched well with the total nurse population in one of the participating hospitals in terms of gender distribution, mean age, and postgraduate specialization: 8.4% men, age 42.7, and 41.3% postgraduate specialists. The gender distribution among bachelor students in nursing at the university colleges is about 10% men. Less than 5% of the potential number of hospital nurses (104 of 2300) participated, while the proportion from primary health care was 20% (99 of 500). If only the best nurses participated, this may partly explain the better results for hospital nurses.

Finally, multiple analyses increased the risk of type 1 errors, but since most of the statistically significant p-values were <0.001, the risk is rather small.

### Implications for practice

The recognition of the nurses' lack of knowledge, particularly in drug management, should be taken into consideration when revising the curriculum in nursing education, and training at work. The quality of existing courses may also be questioned, since courses are demonstrated to be of very small relevance to the knowledge. The institutions, who have legal responsibility for their employers' competence, should emphasize validation of both running and planned courses in medication topics. It is necessary to focus on both the content and regularity of medication courses, and consider some kind of certification for critical areas.

It is of interest to investigate further what happens with certainty and risk of error after continuous courses; whether the risk actually increases in individuals with a high sense of coping. Other possible predictors for high knowledge and certainty will also be of interest to explore, since the background characteristics recorded explain a limited part of the variation in the results. Another aspect that has not been evaluated in this study, is how interruptions influence the risk of errors. Studies have identified distractions and interruptions as the most common contributing factors to medication errors [27,28].

## Conclusions

This study shows that medication knowledge is unsatisfactory among practising nurses, with a significant risk for medication errors. To improve patient safety it is important to take into account several complex mechanisms, and the results have highlighted the need for strengthening nurses' basic knowledge particularly in drug management.

## Competing interests

The authors declare that they have no competing interests.

## Authors' contributions

BS was involved in the design making, responsible for drafting study protocol and tests, performed the data collection, drafting of the analyses, and manuscript. IJ has supervised the study, and contributed with substantial and useful comments and input during all phases. GD has contributed to the planning of the study tests and data collection, given valuable input to the interpretation of the results, and participated in drafting and revisions of the manuscript. LO has contributed to recruit a substantial part of the participants, data collection and critical revision of the manuscript. PF has been project leader and supervised the study, and has made incalculable contributions during all phases. All authors approved the published version.

## Pre-publication history

The pre-publication history for this paper can be accessed here:

http://www.biomedcentral.com/1472-6963/11/175/prepub

## Supplementary Material

Additional file 1 **Multiple Choice Questions - English translation**. The file contains all the questions in the MCQ test, translated into English. The translation is not validated. Click here for file
