# Supplementary material for: Medication knowledge, certainty, and risk of errors in health care: a cross-sectional study
Source: BMC Health Serv Res. 2011 Jul 26;11:175. doi: 10.1186/1472-6963-11-175 (PMC3162500; doi:10.1186/1472-6963-11-175)
Supplement: Additional file 1 — Multiple Choice Questions - English translation. The file contains all the questions in the MCQ test, translated into English. The translation is not validated. [file 1472-6963-11-175-S1.DOC]

For each question, the participants should tick off for one of the alternative answers and also answer the following question: “How certain are you that your answer is right – i.e. what would you do in a real situation?”

0= Very uncertain - would search for help; consulted colleagues/reference books.

1= Relatively uncertain – would probably search for help; consulted colleagues/reference books

2= Relatively certain - would probably not search for help by consulting colleagues/reference books

3= Very certain - would not search for help by consulting colleagues/reference books

**PHARMACOLOGY**

**General pharmacology**

1. Which information do you get from the half-life of a drug?

⁯ The amount of drug given

⁯ The concentration of the drug in the urine

⁯ The elimination rate of the drug

2. What does the pharmacodynamics of a drug mean?

⁯ The effect of the drug on the body

⁯ The fate of the drug in the body

⁯ The drug's affinity to a receptor

3. What do we understand by the term therapeutic index of a drug?

⁯ The ratio of the lethal to the therapeutic dose

⁯ The relationship between the therapeutic dose and the toxic dose of a drug.

⁯ The effect of antibiotics on many types of microorganisms

**Effects**

4. What are the most efficient drugs to obtain bronchodilatation in patients with chronic obstructive pulmonary disease (COPD)?

⁯ Beta 1-stimulators

⁯ Beta 2-stimulators

⁯ Beta-blockers

5. What is the indication for using corticosteroids in rheumatoid arthritis (RA)?

⁯ They reduce inflammatory responses

⁯ They have a positive effect on the immune system

⁯ They cure the disease

6. When is anti-diabetics in oral formulation used?

⁯ If the patient has reduced uptake of glucose from the intestine

⁯ If the patient to a certain extent produces insulin herself

⁯ If the patient breaks down the insulin in the absorption process in the GI tract

**Side effects**

7. Why is it important for asthmatic patients to rinse their mouth after inhalation of corticosteroids?

⁯ In order to prevent fungal growth in the mouth

⁯ In order to prevent atrophy of the mucosa in the mouth

⁯ In order prevent ulcerations in the mouth and throat.

8. What could be a troublesome side effect of many psychotropic drugs?

⁯ Oedema

⁯ Headaches

⁯ Dry mouth

9. What is the most common side effect of nitro-glycerine?

⁯ Headaches, flushing

⁯ Abdominal pain

⁯ Allergic reaction

**Interactions**

10. Which one of these drugs causes the greatest risk for interaction with other drugs?

⁯ Anticoagulants (eg. warfarin)

⁯ Pain killers (eg. paracetamol)

⁯ Tranquilizers (eg. diazepam)

**Administration forms**

11. Why should slow-release tablets be swallowed whole, and not be crushed or cut?

⁯ Because of bad taste

⁯ Because it gives poorer effect

⁯ Because it increases the risk of side effects

12. Many drug name suffixes tell if the drug can be crushed or not. Which one of these drugs can be crushed, by the name of it?

⁯ Lescol Depot

⁯ Moduretic mite

⁯ Cardizem UNO

**Synonymous drugs (generics)**

13. What is a synonymous (generic) drug?

⁯ A drug that is produced in a low-cost country and imported to Norway

⁯ A drug containing the same active ingredients as an original product

⁯ A drug used in addition to an original product

14. Zocor is a lipid-lowering drug, which for a period of time was one of the world’s best selling drugs. Which drug name is synonymous (generic) to Zocor?

⁯ Lipitor

⁯ Pravastatin

⁯ Simvastatin

| **Drug management**  **Liability** |
| --- |

1. Who is legally responsible for organizing the drug management system in hospital / nursing home?

⁯ Head of Department

⁯ Head nurse

⁯ Head of Institution/managing director

2. Who is responsible for signing the medical order from the department to the pharmacy?

⁯ The chief physician

⁯ A physician

⁯ Medicine storage Manager (nurse)

**Storage**

3. The temperature in the drug refrigerator should be between

⁯ 1 to 4 ˚ C

⁯ 2 to 8 ˚ C

⁯ 4 to 8 ˚ C

4. Which one of these drugs should be stored in a poison (narcotics) cabinet?

⁯ Codeine tablets

⁯ Pethidine suppositories

⁯ Diazepam injection

**Durability**

5. For how long time after first opening may normally a sterile, preserved drug in a glass vial be used when stored at room temperature?

⁯ 72 hours

⁯ 2 weeks

⁯ 4 weeks

6. For how long can an insulin pen be stored at room temperature after first opening?

⁯ 2 weeks

⁯ 4 weeks

⁯ 6 weeks

**Preparing for use**

7. Which infusion solution would you choose for Potassium Chloride ?

⁯ Sodium chloride

⁯ Glucose

⁯ Sterile water

8. A patient should have 15 ml liquid laxative, Laktulose. How would you dispense the dose?

⁯ Fill a small medicine cup (30 ml) half full

⁯ Use a 20 ml syringe with needle (pull 15 ml)

⁯ Use a tablespoon

9. A patient has a dry, scaly eczema, and the physician has prescribed cortisone dermally, without further specification. What would you suggest?

⁯ Cortisone solution

⁯ Cortisone cream

⁯ Cortisone ointment

10. A patient has vomited and an antiemetic agent has been prescribed, without specification of administration form. What would you suggest?

⁯ Tablet

⁯ Suppository

⁯ Drug for intravenous injection

**Administration to patient**

11. When should the medication “Kardex” be signed by two persons (double control) when using an injection pump?
⁯ At the start-up

⁯ When changes

⁯ Both at start-up and when changes

12. A patient with heart failure is reluctant to take the nitro-product you offer her, because its appearance is unknown to her. What do you do?

⁯ Reassure her that equal drugs may have different looks when coming from another manufacturer.

⁯ Look up in the Drug Index and check if there are generic products.

⁯ Find the original package in order to check whether the dispensed drug is correct

13. A patient often refuses to take her medicines. What would you do?
⁯ Wait a bit, and then try again or leave it to a collegue to attempt.
⁯ Mixing the drug with jam, so that the patient gets the drug anyway
⁯ Let the patient off, and mark the omitted dose in the drug kardex

14. What is the minimum requirement by Regulations for double control of administration of drugs to patients?

⁯ Always

⁯ When administering addictive drugs, tranquilizers/sedatives

⁯ When administering drugs for injection or infusion

**Drug dose calculations**

1. 1 hour 3 minutes = ⁯ 103 minutes

⁯ 33 minutes

⁯ 63 minutes

⁯ 73 minute

2. 20 micrograms = ⁯ 0,02 mg

⁯ 20000mg

⁯ 0,2 mg

⁯ 0,002 mg

3. Potassium cloride for infusion has the concentration 1 mmol/ml. The physician has prescribed a dose of 25 mmol for infusion. How many ml of the infusion concentrate equals 25 mmol?

⁯ 1 ml

⁯ 250 ml

⁯ 25 ml

⁯ 2,5 ml

4. The patient should have 10 500 IE Heparin as intervenous infusion. The concentration in the vial is 5 000 IE/ml. How many ml do you pull out from the vial?

⁯ 2,1 ml

⁯ 3 ml

⁯ 0,5 ml

⁯ 21 ml

5. 2,5% = ⁯ 250 mg/ml

⁯ 2,5 mg/ml

⁯ 0,25 mg/ml

⁯ 25 mg/ml

6. 250 mg/ml = ⁯ 25 %

⁯ 2,5 %

⁯ 0,25 %

⁯ 250 %

7. 0,42 l = ⁯ 420 ml

⁯ 42 ml

⁯ 0,42 ml

⁯ 4200 ml

8. One Marevan tablet contains 2,5 mg warfarin, og may be divided into 4 pieces. How many mg does a patient get when given 2 and ¼ tablet?

⁯ 1,1 mg

⁯ 1,4 mg

⁯ 5,6 mg

⁯ 6 mg

9. You give a patient 3 and ½ tablet of a drug, and each tablet contains 5 mg. How many mg does the patient get?

⁯ 1,4 mg

⁯ 17,5 mg

⁯ 1,75 mg

⁯ 15 mg

1. Doxorubicin 50 mg injection substance is diluted in 25 ml sterile water. What is the concentration of the solution?

⁯ 1250 mg/ml

⁯ 2 mg/ml

⁯ 0,5 mg/ml

⁯ 20 mg/ml

11. Furadantin tablets contain 5 mg/tablet. The dosage is 3 mg/kg body weight per 24 hours, in two divided daily doses. The child’s weight is 20 kg. How many tablets should the child get each time?

⁯ 0,5 tablet

⁯ 12 tablets

⁯ 6 tablets

⁯ 3 tablets

12. A patient should have 500 ml Glukose 50 mg/ml intravenously. How many ml/hour should the infusion pump be set at, if the infusion time should be 4 hours?

⁯ 125 ml/hour

⁯ 100 ml/hour

⁯ 12,5 ml/hour

⁯ 2,1 ml/hour

13. A patient gets Invertose 120 mg/ml. Due to the risk of acidosis, the infusion rate must not exceed 10 mg/kg/hour. What is maximum drop rate (drops/hour) for a patient weighing 30 kg. The drop number is 20/ml.

⁯ 100 drops/hour.

⁯ 18 drops/hour.

⁯ 60 drops/hour.

⁯ 50 drops/hour.

14. 20 ml Hibitane 20% should be diluted to a solution with the concentration 5 mg/ml. How many ml is the diluted solution?

⁯ 780 ml

⁯ 800 ml

⁯ 80 ml

⁯ 820 ml
